# A phase II study of irinotecan plus chronomodulated oxaliplatin, 5-fluorouracil and folinic acid in advanced colorectal cancer patients

**DOI:** 10.1038/sj.bjc.6601382

**Published:** 2003-11-11

**Authors:** C Garufi, E Bria, B Vanni, A M R Zappalà, I Sperduti, E Terzoli

**Affiliations:** 1 Oncologia Medica C, Istituto Regina Elena, Via E Chianesi 53, 00128 Roma, Italy; 2 Unità Biostatistica, Istituto Regina Elena, Roma, Italy

**Keywords:** colorectal cancer, irinotecan, oxaliplatin, chronotherapy

## Abstract

The combination of irinotecan (CPT-11), oxaliplatin (L-OHP), 5-fluorouracil (5-FU) and folinic acid (FA) is one of the possibilities to overcome chemoresistance in advanced colorectal cancer (ACRC) patients. The aim of this study was to determine the tolerability and activity of CPT-11 plus chronomodulated infusion of L-OHP, 5-FU and FA in ACRC patients. A total of 35 patients (91% pretreated, 77% with CPT-11, 54% with L-OHP, 42% with both) were treated every 3 weeks with CPT-11, 180 mg m^−2^ day 1 i.v., plus L-OHP, 20 mg m^−2^ day^−1^, 5-FU, 700 mg m^−2^ day^−1^ and FA, 150 mg m^−2^ day^−1^, all three drugs from day 2 to day 5 by chronomodulated infusion. The patients' (pt) data were as follows: male/female 21/14; median age 58 years (range: 38–70); PS 0: 26 pts (74%), PS 1: 8 pts (23%), PS 2: 1 pt (3%); primary tumour colon/rectum 26/9; involved organs: 1, 14 pts (40%); 2, 17 pts (48%); ⩾3: 4 pts (11%); previous chemotherapy lines 1: 12 pts (34%), 2: 10 pts (28%), ⩾3: 10 pts (28%). A total of 221 courses (c) were performed; no grade 4 toxicity was observed with only one grade 3 (G3) neutropenia and thrombocytopenia (3%) in one out of 221 courses (<1%). Maximal toxicity (G3) was nausea and diarrhoea in 10 pts (28%), occurring in 14 out of 221 c (6%) and 12 out of 221 c (5%) respectively. Seven patients achieved a partial response (20%, confidence interval (c.i.) 6.8–33.3) and one patient a complete response (2.9%, c.i. 0–8.4), for a total overall response rate of 22.9% (c.i. 9–36.8); 15 out of 35 (42.9%, c.i. 26.5–59.3) had stable disease and 12 out of 35 (34.3%, c.i. 18.6–50) patients underwent a progression. In conclusion, this four-drug regimen is feasible in advanced pretreated ACRC patients with no significant haematological toxicity and acceptable diarrhoea. The activity of this combination is currently studied in EORTC 05011 study.

During the last decade, the introduction of new drugs such as irinotecan (CPT-11) and oxaliplatin (L-OHP) modified the management of metastatic colorectal cancer (CRC). Both drugs had shown activity as a single agent and in combination with 5-fluorouracil (5-FU), with a different mechanism of action and without cross-resistance: SN 38, the active metabolite of CPT-11, inhibits DNA topoisomerase I. L-OHP induces the formation of DNA adducts, inhibiting DNA synthesis.

In the last few years, prospective randomised phase III trials comparing both continuous infusion and bolus 5-FU plus folinic acid (FA) *vs* the same schedule plus CPT-11 or L-OHP showed significant increase of response rate (RR), time to progression (TTP), overall survival (OS), no quality of life (QoL) detriment with acceptable toxicity profile (De Gramont *et al*, 2000; Douillard *et al*, 2000; Saltz *et al*, 2000; Goldberg *et al*, 2002).

These results suggest that combination of infusional 5-FU plus L-OHP or CPT-11 can now be considered as the standard treatment in this setting of patients.

Drug delivery according to circadian rhythm showed an impact on toxicity profile and activity both in preclinical and in clinical setting (Lévi, 2001). It was demonstrated in phase III trials that the combination of L-OHP plus 5-FU and FA delivered by chronomodulated infusion, the FFL regimen, provides more activity and a better toxicity profile when compared to the flat infusion of the same drugs with a five-fold greater mucositis rate due to 5-FU and the double of peripheral neurotoxicity due to L-OHP in the constant infusion regimen (Lévi *et al*, 1994, 1997). Circadian changes in drug pharmacokinetics, target tissue susceptibility, bone marrow DNA synthesis, 5-FU metabolic and catabolic enzymatic activity (thymidylate phosphorylase and dehydropyrimidine dehydrogenase) and oral/rectal epithelium during night hours *vs* light hours are some of the explanations of these differences. Concerning drug activity 5-FU dose-intensity seems to be related to drug activity and this could influence survival (Lévi *et al*, 1999; Garufi *et al*, 1997).

The combination of CPT-11 and L-OHP without 5-FU was also investigated in phase I and II studies, which showed the activity of these drugs in patients heavily pretreated with 5-FU-based regimen (Scheiteuer *et al*, 1999; Wassermann *et al*, 1999). Haematologic toxicity has been commonly observed; grade 3–4 (G3–4) neutropenia occurred in 20–33% of patients even though concomitant granulocyte colony-stimulating factor (G-CSF) administration. Synergistic *in vitro* experimental evidence of activity between these two drugs were found: pre-exposure to L-OHP enhances the cytotoxic effects of SN38 by increasing topoisomerase I activity by a stabilisation of DNA platinum adducts, the duration and extent of DNA and RNA synthesis inhibition and through a prolonged DNA elongation inhibition (Zeghari-Squalli *et al*, 1999). *In vitro* model on CRC cell lines had suggested that the activity of SN-38 is synergic to L-OHP plus 5-FU-FA when delivered 24–48 h before.

The preclinical data cited above strongly support the attempt to overcome the chemoresistant clones using all active drugs together with an acceptable cost in terms of patient tolerability. Now, we present the results of a phase II trial designed to determine the toxicity profile and activity of the combination of CPT-11 plus L-OHP, 5-FU and FA by chronomodulated infusion in heavily pretreated metastatic CRC patients.

## MATERIALS AND METHODS

### Patient eligibility

Histologically proven locally advanced or metastatic colorectal cancer; age at least 18 years old; Performance status (World Health Organization) 0–2; measurable disease; life expectancy of at least 3 months; adequate haematologic parameters (white blood cells, WBC ⩾3.5 × 10^9^ l^−1^, absolute neutrophil count ⩾1.5 × 10^9^ l^−1^, platelets ⩾100 × 10^9^ l^−1^, haemoglobin ⩾10g dl^−1^); bilirubin ⩽1.25 × upper normal limit (UNL); alanine aminotransferase (ALT) and aspartate aminotransferase (AST) ⩽2.5 × UNL; or bilirubin ⩽1.5 × UNL, ALT and AST ⩽5 × UNL in patients with liver metastases; serum albumin ⩾3 g l^−1^; and normal renal function, with serum creatinine ⩽1.25 × UNL and creatinine clearance of at least 60 ml min^−1^; previous chemotherapy for metastatic disease was allowed. Patients were considered not eligible for the following reasons: secondary primary tumour other than nonmelanoma skin cancer or *in situ* cervical carcinoma; metastatic lesion suitable for surgical resection of elective radiotherapy; inflammatory bowel diseases or chronic diarrhoea that requires treatment; total colectomy or ileostomy; bowel obstruction and/or subobstruction; severe diarrhoea (WHO G3–4) during prior 5-FU or CPT-11 chemotherapy; uncontrolled metabolic disorders or active infections; uncontrolled cardiac arrhythmias; uncontrolled congestive heart failure or severe ischaemic heart disease; acute myocardial infarction in the last 6 months; history of significant neurologic or psychiatric disorders; pregnancy or breastfeeding; symptomatic brain metastases; prior irradiation affecting more than 30% of active bone marrow; ongoing treatment with other antiblastic agents or radiotherapy; functional grade 3 (G3) neuropathy due to L-OHP.

### Pretreatment evaluation

Each patient had to sign written informed consent to enter the study. Every patient was required to have a totally implanted, double-lumen, central venous access port. Within 4 weeks before starting the treatment, patients were required to perform baseline imaging work-up by thorax, abdomen and pelvis CT scan; initial blood sample not far than 1 week prior to start chemotherapy. Patients had to be completely out of toxic effect of previous treatment.

### Chemotherapy

The schedule we applied in this study is based on the following: (a) L-OHP 20 mg m^2^ day^−1^ plus 5-FU 600 mg m^2^ day^−1^ and FA 150 mg m^2^ day^−1^ by chronomodulated infusion were the starting doses in the 3-weekly FFL schedule used in our phase III trials (Lévi *et al*, 1994, 1997); (b) we previously conducted a phase II study where CPT-11, 180 mg m^−2^ on day 1 in 1 h by a 6-h chronomodulated infusion, was combined to chronomodulated 5-FU and FA from day 2 to day 5 (Garufi *et al*, 2002). To increase the activity of this last combination, L-OHP at the dose of 20 mg m^2^ die^−1^ (days 2–5), was added to the previous CPT-11 (day 1) plus the 4-day (days 2–5) 5-FUFA chronomodulated infusion every 3 weeks.

**Figure 1:**
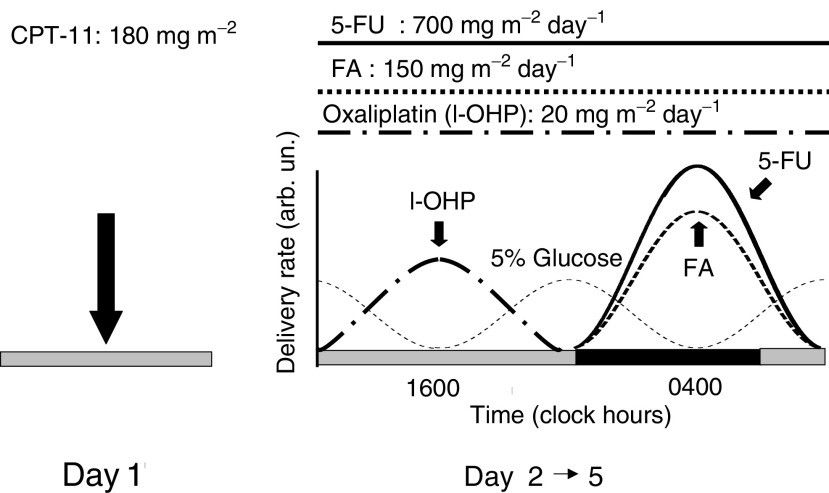
Treatment schedule (for 5 days every 3 weeks).

CPT-11 was administered during the morning as a 60 min intravenous (i.v.) infusion, dissolved in 250 ml of 0.9% NaCl solution, on day 1 at the dose of 180 mg m^−2^. Atropine was given before CPT-11 to avoid cholinergic syndrome and loperamide was suggested for delayed diarrhoea. L-OHP, 5-FU and FA were administered by chronomodulated infusion using a multichannel, programmable, in-time, ambulatory pump, equipped with four channels (Melodie® pump). Pumps were programmed using Aguettant software. L-OHP infusion was administered from 1000 to 2200 with a diurnal peak at 1600 for 4 consecutive days (days 2–5) at the dose of 20 mg m^−2^ day^−1^ (days 2–5). 5-FU and FA infusions were both delivered the same days from 2200 to 1000 with a nocturnal peak at 0400 at the dose of 700 mg m^−2^ day^−1^ (5-FU) and FA was administered at the fixed dose of 150 mg m^−2^ day^−1^ (Figure 1

). Antiemetic prophylaxis with anti-HT-3 was administered to each patient. No steroid medication was allowed. Toxicity was evaluated after each course during treatment according to WHO criteria. Treatment was planned every 3 weeks, depending on patient compliance and tolerance to treatment.

### Dose modifications

*Diarrhoea*: CPT-11 dose was reduced to 130 mg m^−2^ and 5-FU dose by 100 mg m^−2^ day^−1^ in the presence of G3–4 diarrhoea. *Mucositis*: 5-FU was reduced by 100 mg m^−2^ day^−1^ in the presence of G3–4 mucositis or cutaneous toxicity. *Neutropenia*: In case of absolute neutrophil count ⩽1.5 × 10^9^ l^−1^ and/or platelet count ⩽100 × 10^9^ l^−1^, treatment had to be delayed for 2 weeks maximum. No G-CSF administration was allowed unless necessary. *Neuropathy*: L-OHP was reduced to 15 mg m^−2^ day^−1^ in case of persistent (<14 days) paresthesia or occurrence of functional impairment; ⩾14 days provided L-OHP discontinuation.

### Patient evaluation

Tumour response were assessed using World Health Organization criteria as follows: complete response (CR) was considered the complete disappearance of all clinical palpable, endoscopical and imaging evidence of tumour, determined by two observations not less than 28 days apart. Partial response (PR) was defined as a reduction greater or at least equal to 50% of the sum of the products of the two longest diameters measured lesions. No increase in the size of any lesion or appearance of new lesions must have occurred. Stable disease (SD) was defined as a reduction less than a PR, but without evidence of disease progression. Progression of disease (PD) was defined as an increase of at least 25% in the product of measured lesion and/or appearance of new lesion while the patient was on study. CT scanning was repeated every 4 weeks in case of CR or PR to confirm the response. The TTP was defined as the interval between the first treatment and the first documentation of disease progression. Time to treatment failure (TTF) was defined as the interval between the first and the last day of treatment. Duration of response was considered as the time between first appearance of response and tumour progression. Overall survival was measured from the time of starting treatment to death or last contact with the patient.

## RESULTS

### Patient characteristics

From September 1999 to May 2002, 35 patients affected by metastatic colorectal cancer entered the study. Patients' characteristics are listed in Table 1

**Table 1:** Patient characteristics

	**No. of patients (total: 35)**	**%**
*Age (years)*		
Median (range)	58 (38–70)	
		
*Gender*		
Male	21	60
Female	14	40
		
*Performance status (WHO)*		
0	26	74
1	8	23
2	1	3
		
*Primary tumour*		
Colon	26	74
Rectum	9	26
		
*Site of metastasis*		
Liver	26	74
Lung	18	51
Lymph nodes	6	17
Peritoneum	5	14
Other	4	11
		
*No. of organs involved*		
1	14	40
2	17	48
⩾3	4	11
		
*Radiotherapy*		
Adjuvant	4	11
Palliative	6	17
		
*No. of previous chemotherapy lines*		
1	12	34
2	10	28
⩾3	10	28
		
*5-Fluorouracil-based chemotherapy*		
Adjuvant	12	34
Palliative	32	91
		
*Previous chemotherapy*		
L-OHP	19	54
CPT-11	27	77
L-OHP+CPT-11	15	42
		
*Baseline CEA*		
Median (range)	35.5 (1–7586)	

. The median age was 58 years (range 38–70), 26 patients (74%) had WHO performance status of 0, 26 patients (74%) had colon as primary sites of disease and the liver was the site of distant metastases in 26 patients (74%). In all, 11 patients (31%) had metachronous metastases and 24 (69%) had synchronous distant metastasis. Out of 35 patients, 32 (91%) had received first-line chemotherapy: all of these patients were pretreated with 5-FU-based chemotherapy; 27 patients (77%) with CPT-11 (median dose received 858 mg m^−2^), 19 patients (54%) with L-OHP (median dose received 230 mg m^−2^), 15 patients (42%) had previously received both CPT-11 and L-OHP.

### Treatment toxicity

A total of 221 courses were administered, with a median number of five courses (range 2–15). Median CPT-11 dose-intensity was 48.5 mg m^2^ week^−1^ (80.8% of the theoretical dose of 60 mgm^2^ week^−1^); median L-OHP dose-intensity was 24.4 mg m^2^ week^−1^ (89.9% of the theoretical dose of 26.7 mg m^2^ week^−1^); median 5-FU dose-intensity was 840 mg m^2^ week^−1^ (90% of the theoretical dose of 933.3 mg m^2^ week^−1^). All patients were assessable for toxicity. The overall toxicity per patient and per course are shown in Table 2

**Table 2:** WHO toxicity criteria in 35 patients and 221 courses

	**Grade**							
	**1**		**2**		**3**		**4**	
	**Patients/courses**	**%**	**Patients/courses**	**%**	**Patients/courses**	**%**	**Patients/courses**	**%**
*Haematologic toxicity*								
Anaemia	3/13	8.5/6	3/3	8.5/1	—	—	—	—
Leucopenia	12/28	34/12.5	4/7	11.5/3	1/—	3/—	—	—
Neutropenia	10/26	28.5/11.5	4/7	11.5/3	1/1	3/0.5	—	—
Thrombocytopenia	—/1	—/0.5	1/1	3/1	1/1	3/0.5	—	—
								
*Nonhaematologic toxicity*								
Nausea	7/55	20/25	19/78	54/35	10/13	28.5/6	—	—
Vomiting	9/63	25.5/28,5	14/31	40/14	8/8	23/3.5	—	—
Diarrhoea	10/53	28.5/24	11/29	31.5/13	10/11	28.5/5	—	—
Mucositis	6/17	17/7.5	6/10	17/4.5	3/3	8.5/1	—	—
Cutaneous	5/14	14/6	—	—	—	—	—	—
Neurotoxicity	7/60	20/27	2/6	6/2.5	—	—	—	—
Asthenia	6/66	17/29.5	22/41	63/18.5	4/6	11.5/2.5	—	—

, respectively. Neither febrile neutropenia was registered nor any other grade 4 (G4) toxicity occurred. The most serious G3 toxicities were nausea and diarrhoea, which occurred in 10 of 35 patients (28.5%). G3 vomiting was present in eight patients (23%). The most frequent G3 toxicities was nausea, occurred in 13 out of 221 courses (6%), diarrhoea, 1 out of 221 (5%) and vomiting, eight out of 221 (3.5%). Four of 35 patients (11.5%) had G3 asthenia, which occurred in six out of 221 courses (2.5%).

### Treatment efficacy

All patients were evaluable for response. Seven patients achieved PR (20%, confidence interval c.i. 6.8–33.3) and one patient CR (2.9%, c.i. 0–8.4), for a total overall response rate (ORR) of 22.9% (c.i. 9–36.8); 15 patients (42.9%, c.i. 26.5–59.3) had SD. A total of 12 patients (34.3%, c.i. 18.6–50) deemed in progression (Table 3

**Table 3:** Overall response rate (ORR)

	**ORR (*n*/%)**	**CR (*n*/%)**	**PR (*n*/%)**	**SD (*n*/%)**	**PD (*n*/%)**
All evaluable patients (*n*=35)	8/22.9%	1/2.9%	7/20%	15/42.9%	12/34.3%
(intention to treat analysis)	(c.i. 9–36.8)	(c.i. 0–8.4)	(c.i. 6.8–33.3)	(c.i. 26.5–59.3)	(c.i. 18.6–50)
					
Patients not pretreated with CPT-11 or L-OHP (*n*=4)	1	1	3	—	—
Patients pretreated with CPT-11 (*n*=12)	3	—	3	4	5
Patients pretreated with L-OHP (*n*=4)	1	—	1	3	—
Patients pretreated with both CPT-11 plus L-OHP (*n*=15)	—	—	—	8	7

CR=complete response; PR=partial response; SD=stable disease; PD=progression of disease; CPT-11=irinotecan; L-OHP=oxaliplatin.

). The median time of response duration was 4.5 months.

### Efficacy and previous treatment

All the four CPT-11 and L-OHP untreated patients obtained an objective response (one CR and three PR). One out of the four L-OHP (25%) pretreated patients achieved a PR and three had SD. A total of 12 patients had previously received only CPT-11 without L-OHP: three of them (25%) had PR, four had SD, and four patients underwent progressive disease. None of the 15 patients previously treated with both CPT-11 and L-OHP achieved tumour reduction; eight of them had SD and seven had progression of disease. Among the six patient responders at the previous chemotherapy line, three of them had PR (50%), one had no change of disease and two had progression. A total of 13 patients had no change of disease at the previous chemotherapy: nine of them maintain SD, one had PR and three deemed in progression. Out of the 10 progressive patients at the previous chemotherapy line, five achieved no change of disease and five kept progressing. The median TTF was 3.5 months (c.i. 3–5). The median TTP and OS were 4.1 months (c.i. 4–7) and 15.1 months (c.i. 10–18) respectively, with a median follow-up of 8.5 months.

### Efficacy and surgery

Four patients underwent surgery after chemotherapy. Two patients who achieved PR after seven courses of treatment underwent lung and liver metastasectomy, respectively; the patients with hepatic resection received radical surgery and received further four courses of the treatment. Two patients with SD underwent surgery after chemotherapy; a patient received radical lung metastasectomy and the other underwent cerebellar resection for brain metastasis 3 months after progression of disease. Another patients who obtained SD underwent liver termoablation of metastases; the patient deemed in progression after further 3 months. The majority of the patients who were considered suitable for further chemotherapy received a combination containing mitomycin-C plus UFT or 5-FU continuous infusion.

## DISCUSSION

The treatment of advanced colorectal cancer changed profoundly during the last 10 years. The best results in terms of RR, time to progression and OS were obtained when infusional 5-FU and leucovorin were combined to mitomycin-C, oxaliplatin or CPT-11. At this moment, there is no standard rule regarding the optimal dose and schedule of both 5-FU and oxaliplatin, while the use of CPT-11 seems to be preferred in combination with FA and infusional 5-FU (Douillard *et al*, 2000).

The chronomodulation of 5-FUFA plus L-OHP (FFL), developed by Lévi and tested in several phase II and III trials, contributed to the definition of a new strategy in this disease, including the reduction of 5-FU and L-OHP toxic effects and the possibility to use chemotherapy as a neoadjuvant therapy for advanced patients in order to increase the number of patients suitable for surgery after chemotherapy (Adam *et al*, 2000). So the next step was to improve these results: the logical consequence was to add CPT-11 to the chronomodulated schedule of FFL.

From our point of view, the addition of CPT-11 to our standard treatment, which is the chronomodulated regimen of FFL, is justified if a synergistic rather than an additive effect can be produced in order to avoid severe toxic effects in the majority of patients. In this context, our group previously demonstrated in an experimental mouse model that the synergy between CPT-11 and L-OHP is obtained only when the two drugs are given at their optimal timing, which is during the second half of activity span for L-OHP and during the second half of rest span for CPT-11, coming to a peak time of 1600 for L-OHP and of 0500 for CPT-11 in human beings (Granda *et al*, 2002). At this time, there is no clear evidence that CPT-11 chronomodulation can help to significantly increase effectiveness or tolerability of this drug, when it is used alone or in combination with 5-FU and it is compared to the standard 1-h infusion regardless of peak timing (Garufi *et al*, 2002; Giachetti *et al*, 2001).

In order to test the feasibility and tolerability of this new regimen, we tested 35 pretreated colorectal cancer patients. Our results pointed out two points: the schedule we proposed is feasible with an acceptable toxicity, mainly diarrhoea and nausea. It is crucial to observe that we did not use steroids for nausea and vomiting prevention because of their negative effects on circadian coordination. The second point is that there is no activity in patients previously treated separately with both CPT-11 and L-OHP and 5-FU, and so the use of this four-drug combination in this set of patients seems not to be justified as already found when patients become resistant to combination of oxaliplatin and infusional 5-FUFA (Andrè *et al*, 1999; Tournigaud *et al*, 2001).

Souglakos (Souglakos *et al*, 2002) and Falcone (Falcone *et al*, 2002) recently published the first two trials with the four-drug regimen with two different schedules in untreated patients. There are some peculiar differences in patientcharacteristics, tolerability and tumour response between those trials and this one. Both of these authors treated naïve patients so the response rate was 58.1 and 69%, respectively but their schedules, given every 2 weeks, produced substantially toxicity. Souglakos reported 32% of patients with diarrhoea but 45% of them displayed severe G3–4 neutropenia with 6% of febrile neutropenia. Falcone also declared 14% of patients with febrile neutropenia, 55% of them experiencing at least one episode of G4 neutropenia and 21% G3 diarrhoea. In this last trial, a semi-intermittent circadian delivery of 5-FU was employed. Our major toxic effect was G3 diarrhoea and nausea in 28.5% of patients but only 5 and 6% of 221 courses, respectively.

Following the present experience and the reported experimental data, the EORTC Chronotherapy Group is actually conducting a trial in advanced colorectal cancer patients with the four-drug combination where patients are randomised to six different peak timings of CPT-11 on day 1 and receive chronomodulated FFL from day 2 to day 5 every 3 weeks (EORTC 05011). This trial will produce definitive clarification not only on the CPT-11 chronomodulation but also on the activity of this combination in naïve patients.

In conclusion, the present paper showed that the addition of CPT-11 to chronomodulated FFL is feasible with acceptable toxicity in heavily pretreated patients. The activity of this schedule and the role of CPT-11 chronomodulation is actually tested in EORTC 05011 trial in naïve patients.
